# General practice in an integrated NHS: Reasons to be hopeful

**DOI:** 10.1016/j.fhj.2024.100144

**Published:** 2024-05-17

**Authors:** Manpreet Kaur, Christy Chan, Priyankaa Mistry, Harish Vamadevan, Katherine Qualey, Pippa Oakeshott

**Affiliations:** Population Health Research Institute, St George's, University of London, London, SW17 0RE, United Kingdom

The NHS is struggling with increased workload, funding deficits and a shortage of GPs, many of whom ‘are looking for a dignified exit from the profession they once loved.’^1^ Despite this, Beresford and colleagues identify reasons to be hopeful.^1^ One reason is the establishment of integrated care systems which may help reduce professional tribalism and champion disease prevention. Another is that general practice is a trailblazer with clinical coding during consultations ‘generating a huge dataset with remarkable research potential.’^1^ This makes it relatively simple for medical students to conduct audits in primary care. These may improve clinical practice and encourage students to consider becoming general practitioners.

In 2023 we hosted five medical students in our inner-city practice which has about 10,000 ethnically diverse patients. Each student chose a clinical topic in which they had a particular interest. Three chose to audit management of specific illnesses (chronic kidney disease,^2^ gestational diabetes mellitus^3^ and eczema) and two looked at GP prescribing. With the help of our deputy practice manager, they then collected anonymised practice data and compared patient management with National Institute for Health and Care Excellence (NICE) guidelines (Table 1).

**Table 1:** Medical student audits conducted in 2023–24 at an ethnically diverse inner-city practice.

Topic (Author)	Audit question	Audit findings
**Chronic kidney disease CKD (KQ)**	How many patients have two eGFR<60 and no code of CKD in their record?	64 patients needed to have the CKD code added
**Eczema (MK)**	How many adult patients with mild/moderate eczema were treated according to NICE guidelines?	Of 120 patients, 93% were advised to use emollients, 95% were prescribed mild-moderately potent steroid creams, but only 41% had recorded advice to avoid soap and bubble baths.
**Urinary tract infection UTI (HV)**	How many non-pregnant women aged 18–65 with UTI were prescribed recommended antibiotics?	Most women (74%, 39/53) were prescribed nitrofurantoin, but 40% were given longer than the recommended three-day course
**Gestational diabetes mellitus GDM (CC)**	How many women with a history of GDM have had their HbA1C checked in the past 12 months?	Only 44% (16/36) women with a history of GDM had had their HbA1C checked in the past 12 months.
**Teratogenic drugs (PM)**	How many women of childbearing age who were prescribed teratogenic drugs were advised to use highly effective contraception?	All women aged 16–45 on sodium valproate or topiramate had recorded advice to use effective contraception, but only 64% (7/11) of those on pregabalin.

Conducting these audits gave students an opportunity to work alongside family doctors and healthcare staff in a busy general practice. Discussing and implementing findings with the practice team helped to change practice and improve patient care. For example, having a chronic kidney disease (CKD) diagnosis added to a patient's record should encourage the practice to make sure blood pressure and cholesterol are adequately controlled. (This is important since patients with CKD have an even higher risk of cardiovascular disease than diabetics.) This year another group of students will repeat these audits to complete the cycle and see if they have led to improvements in patient care.

Beresford and colleagues conclude: ‘The NHS is full of brilliant, inspiring people……..who leave us hopeful about the next 75 years.’^1^ Conducting these audits was good for staff and student morale and may benefit patients. Four of the five students are hoping to become general practitioners.

## CRediT authorship contribution statement

**Manpreet Kaur:** Conceptualization, Writing – original draft, Writing – review & editing. **Christy Chan:** Conceptualization, Data curation, Writing – review & editing. **Priyankaa Mistry:** Conceptualization, Data curation, Writing – review & editing. **Harish Vamadevan:** Conceptualization, Data curation, Writing – review & editing. **Katherine Qualey:** Conceptualization, Data curation, Writing – review & editing. **Pippa Oakeshott:** Conceptualization, Writing – original draft.

## Declaration of competing interest

The authors declare that they have no known competing financial interests or personal relationships that could have appeared to influence the work reported in this paper.
